# Impact of Endometriosis-Related Adhesions on Quality of Life among Infertile Women

**DOI:** 10.22074/ijfs.2019.5572

**Published:** 2019-01-06

**Authors:** Azza Ibrahim Abd El-Kader, Amina Saad Gonied, Mohamed Lotfy Mohamed, Sabah Lotfy Mohamed

**Affiliations:** 1 Department of Obstetrics and Gynecological Nursing, Faculty of Nursing, Zagazig University, Zagazig, Egypt; 2 Department of Obstetrics and Gynecology, Faculty of Medicine, Zagazig University, Zagazig, Egypt

**Keywords:** Adhesions, Endometriosis, Impact, Infertility, Quality of Life

## Abstract

**Background:**

Endometriosis is considered the most common cause of pelvic adhesions in women. Endometriosis- associated adhesions could result in the formation of fibrous bands, which contain endometriotic glands, stroma and scarring. The aim of this study was to identify the impact of endometriosis-related adhesions on quality of life among infertile women.

**Materials and Methods:**

This descriptive study was conducted at Endoscopic Unit, in Zagazig University Hospitals, Egypt. Oral consent for participation in this study was taken from 109 women who were candidates for laparoscopy as infertile cases and were diagnosed with endometriosis. They were classified into two groups namely, group I (n=41) who had endometriosis with adhesions and group II (n=68) who had endometriosis without adhesions. A structured interviewing form, adhesion scoring method of the American Fertility Society, and Global Quality of Life Scale were used to collect required information.

**Results:**

The prevalence of adhesions resulted from endometriosis was 37.6%. Demographic characteristics of the women with endometriosis-related adhesions were not significantly different from those of women without endome- triosis-related adhesions. The most common location for endometriotic adhesions was adnexal adhesion (51.2%) fol- lowed by adhesion of anterior abdominal wall (24.4%). Quality of life was significantly impacted by endometriosis- related adhesions (P=0.002).

**Conclusion:**

A high percentage of studied patients had a moderate degree of adhesions. Adhesions caused by endo- metriosis had an impact on quality of life of the studied women.

## Introduction

Endometriosis is a chronic gynecological condition in which, endometrial tissue is found outside the uterine cavity. It is a relatively common disorder among women of reproductive-age and is associated with marked pain and morbidity (1). The definite cause of endometriosis is unknown, but the theory of retrograde menstruation has received the most attention. It causes endometrial tissue to reflux through the uterine tubes into the peritoneum (2).

Endometriosis is considered one of the main underlying causes of the development of adhesions unrelated to a previous operation (3). Adhesions are bands of connective tissue, which connect two different tissues that are normally separated thus, interfering with the function of the organs that are affected (4).

Local inflammation associated with endometriosis is viewed as an important element in the formation of adhesions. Adhesions may form as a result of endometrial implants bleeding into the surrounding area and causing an inflammatory reaction which leads to the formation of a band between two organs. Adhesions correlated with endometriosis have different types (i.e. thin, filmy and transparent or thick, dense, and opaque). In severe cases, adhesions found within the pelvis, could cause a fatal condition called “frozen” pelvis (5).

There are many complications associated with endometriosis- related adhesions such as dyspareunia, and rectal constriction which leads to constipation. Also, in this sense, adhesions correlated with endometriosis, are responsible for infertility, chronic pelvic pain, and bowel obstruction (6).

Women with pelvic adhesions should be informed about the risk associated with endometriosis-related adhesions and instructed how to deal effectively with this condition (7). There are several studies reporting endometriosis- related adhesions that affected women's life physically, mentally and socially in the form of social withdrawal, fatigue, lack of enthusiasm for their work, decreased libido, negative self-image, pessimistic attitude and worthlessness (8).

The study aimed to identify the impact of endometriosis- related adhesions on quality of life among infertile women.

## Materials and Methods

The present descriptive study was conducted to identify the impact of adhesions associated with endometriosis on quality of life among infertile women. The study was performed at Endoscopic Unit, in Zagazig University Hospitals in Egypt. This research was conducted after getting permission from the director of Faculty of Nursing-Zagazig University and director of Endoscopy Unit.

These cases were chosen from more than 756 cases from December 2016 to March 2018. Among 756 cases, only 109 women were candidates for laparoscopy due to infertility issues and were diagnosed with endometriosis. According to the revised American Society for Reproductive Medicine (r-ASRM) classification of endometriosis (9), we recruited all the patients with stage III (moderate) endometriosis. Women were eligible for recruitment in this study if they met the following criteria: married women with primary or secondary infertility, women diagnosed with endometriosis with adhesion based on laparoscopy and also, women who desired to participate in this study.

### Exclusion criteria

i. Existence of adhesions due to other reasons such as pelvic inflammatory disease (PID) or myoma or adenomyosis, ii. Existence of chronic pelvic pain that defined as pelvic pain which is constant or cyclical in nature for 6 months or more, iii. Women with previous surgery in the abdomen or pelvis (cesarean section or appendectomy), and iv. Women with autoimmune and/or allergic disease.

Oral consent was taken from women who desired to participate in this research. All procedures involving human participants were in accordance with the ethical standards of the institutional and/or national research committee as well as the 1964 Helsinki Declaration and its later amendments or comparable ethical standards. The study was approved by the Zagazig University-Faculty of Nursing Ethical Committee with the ethical code ZU.NUR/25/22-8-2016.

After revising the laparoscopic report details for recruited women (n=109), participants were grouped into group I (n=41) with subjects who had endometriosis with adhesions and group II (n=68) with subjects who had endometriosis without adhesions.

The tool used for data collection in this research was a structured interview form which was designed by the researchers in order to gather the following data: demographic characteristics as; age (years), body mass index (BMI) (Kg/m^2^), educational level and occupational status. Also, using this tool, the following variables were recorded: duration of the marriage (years), menarche age (years), contraception type and family history of endometriosis.

Adhesions detected during laparoscopy were divided regarding the locality into adenxal adhesion, anterior abdominal wall adhesion, vesico-uterine adhesion, uterus to abdominal wall adhesion, and frozen pelvis.

Adhesions were classified in terms of severity by grading using adhesion scoring method of the American Fertility Society (AFS) (10): i. Mild adhesions, which are thin, filmy, vascular, and transparent adhesions that are easily cut with blunt dissection and subsequently easily freeing adherent organs, ii. Moderate adhesions, which are opaque, possess moderately thick layers, exert moderate degrees of vascularity and bleed minimally on dissection, and iii. Severe adhesions, which are very thick, opaque, and highly vascular and bleed much on dissection.

Validated language version of the Global Quality of Life Scale (GQOL) was used to measure the quality of life for women with adhesions related to endometriosis. The Global Quality of Life Scale is a single scale that directly evaluates the quality of life by patients themselves by using a scale between 0 (='no quality of life') and 100 (='perfect quality of life'). Also, in this scale, each patient was asked to describe her life quality by writing a number between 0 and 100 (11). Global quality of the life scale is the person's overall judgment of his/her life quality, so we particularly used this tool to assess the impact of adhesions on the women's quality of life who were not able to return to hospital again after performing the laparoscopic procedure. The tool was tested for content validity and reliability by five professors in the field of Obstetrics and Gynecological Medicine.

Data were gathered and outcome measures were coded, entered into and analyzed by Microsoft Excel program. Statistical analysis was done with IBM SPSS Statistics V. 20 (SPSS Inc., Chicago, IL, USA). According to the type of data, qualitative data were presented as number and percentage, continues quantitative data were represented as mean ± SD; also, the following tests were utilized to test differences between groups; difference and association of qualitative variables were tested by Chi-square test (X^2^) and differences between quantitative independent groups were assessed by t test. A P<0.05 was regarded as statistically significant. Also, cases who received a score =40 in global quality of life scale were considered the cases whose quality of life was affected and cases with a score = 45 in global quality of life scale, were considered the cases whose quality of life was not affected. We considered in our study score 40 or less to be impacted on quality of life, according to classification suggested by Hyland and Sodergren (11).

**Table 1:** Demographic characteristics of women with and without endometriosis-related adhesions


Variables		Group I(cases with endometriosis-related adhesions) (n =41)n (%) or (mean ± SD)	Group II (cases without endometriosis- related adhesions) (n=68)n (%) or (mean ± SD)	T test	P value

Age (Y)		32.1 ± 5.6	31.7 ± 5.8	0.34	0.7
Menarche age (Y)		12.2 ± 1.4	12.6 ± 1.3	1.2	0.1
Duration of marriage (Y)		5.8 ± 1.5	5.6 ± 1.7	0.48	0.5
BMI (Kg/m^2^)		30.2 ± 4.5	29.5 ± 4.8	0.737	0.4
				X^2^	
Educational level					
	Elementary education	12 (29.3)	19 (27.9)	0.18	0.9
	Secondary education	12 (29.3)	18 (26.5)		
	College education or above	17 (41.4)	31 (45.6)		
Occupational status					
	Worked	10 (24.4)	16 (23.5)	0.01	0.9
	Not worked	31 (75.6)	52 (76.5)		
Family history of endometriosis	
	No	12 (29.3)	21 (30.9)	0.03	0.8
	Yes	29 (70.7)	47 (69.1)		
Contraceptive type					
	None	31 (75.6)	40 (58.8)	3.8	0.1
	Oral	6 (14.6)	21 (30.9)		
	IUD	4 (9.8)	7 (10.3)		


BMI; Body mass index, IUD; Intrauterine device, T test; Independent samples t test, MCP; P value based on Mont Carlo exact probability, and X^2^; Chi-square test.

## Results

In this study, 109 cases with endometriosis were enrolled at the time of laparoscopy. Among them, 41 participants were found to have adhesions with endometriosis and 68 cases had no adhesions with endometriosis; thus, the prevalence of endometriosis-related adhesions was 37.6%.

The mean and the standard deviation (mean ± SD) of age, BMI, menarche age and duration of marriage of the women with and without endometriosis-related adhesions are presented in Table 1.There was no statistically significant difference in the afore-mentioned factors between the women with adhesions and the women without adhesions (P=0.7, 0.4, 0.1 and 0.5, respectively).

Also, based on data shown in Table 1, we found no correlations between the two groups regarding educational level, occupational status, family history of endometriosis and contraceptive type (P=0.9, 0.9, 0.8 and 0.1, respectively).

Table 2 reveals that the greater part of the studied women (51.2%) had adnexal adhesion followed by anterior abdominal wall adhesion 10 (24.4%) and vesico-uterine adhesion 5 (12.2%). Few patients (2.4%) had frozen pelvis. Concerning the severity of adhesions resulted from endometriosis, Table 2 indicates that almost all of the participated women had a moderate degree of severity 19 (46.3%) and only nine cases (22.0%) showed a severe degree of adhesions.

**Table 2:** Locations and grading of pelvic adhesions (n=41)


Parameters		n (%)

Locations of adhesion		
	Adnexal adhesion	21 (51.2)
	Anterior abdominal wall adhesion	10 (24.4)
	Vesico-uterine adhesion	5 (12.2)
	Uterus to abdominal wall adhesion	4 (9.8)
	Frozen pelvis	1 (2.4)
Grading of the pelvic adhesion		
	Mild	13 (31.7)
	Moderate	19 (46.3)
	Severe	9 (22.0)


Based on Table 3, it was found that the quality of life was significantly impacted by adhesions (P=0.002). Chi-square test (X^2^) showed a significant association between impacted quality of life and adhesions that were resulted from endometriosis as 34.1% of cases with adhesions had a negatively influenced life while only 10.3% of cases without adhesions reported a negatively impacted quality of life.

**Table 3:** Association between adhesions related to endometriosis and quality of life (n=109)


Variable		Group I(cases with adhesions related to endometriosis) (n=41)	Group II (cases without adhesions related to endometriosis) (n=68)	X^2^	P value

Quality of life					
	Not impacted	27 (65.9)	61 (89.7)	9.3	0.002^*^
	Impacted	14 (34.1)	7 (10.3)		


Data are presented as n (%). X^2^; Chi-square test, MCData P; P value based on Mont Carlo exact probability, and *; P<0.05 (significant).

## Discussion

Endometriosis is a condition in which a multiple interplay between the shed endometrial tissue, the peritoneal environment, and the peritoneal lining occurs. When the peritoneum cannot remove the endometrial tissue in time, these tissues will have the chance to adhere to the peritoneal lining which finally leads to this disease (12). Formation of adhesions is a major complication of surgical treatment of endometriosis. Also, recent researches suggest that peritoneal inflammation, which may cause adhesions, occurs in the presence of active endometriosis (13).

Adhesions and endometriosis are connected together because endometriosis is an adhesiogenic disease. The nature of recurrence of endometriosis means that repeated surgical operations are usually performed, which in turn increase the chance of adhesion formation. So, the current research was performed to understand the impact of adhesions with endometriosis on the women’s quality of life.

The prevalence of adhesion among women with endometriosis in the current study, was 37.6%. While, the study conducted by Parker et al. (14) showed that 74.0% of women had pelvic adhesions at the first surgery, and 82.0% of cases had adhesions at the time of second surgery. Of the 8 cases without a previous endometriosis surgery, 6 (75.0%) had adhesions at the first operation.

Concerning the demographic characteristics of patients, the two groups with and without adhesions were similar in age, occupational status, level of education and body mass index. Thus, these variables did not influence the frequency of adhesions in each group, nor the risk factors analyzed in the current study. 

On the other hand, our study did not include any women with an extremely high BMI; mean and the standard deviation (mean ± SD) of BMI were 30.2 ± 4.5 in patients with adhesions associated with endometriosis and 29.5 ± 4.8 in the group of women without adhesions. The above- mentioned results were coinciding with those reported by Stocker et al. (15).

The majority of studies to date, have reported that early menarche (<11 years) increases the danger of endometriosis, but our results did not find any significant dif¬ference between the age of menarche and endome-triosis and the formation of adhesion. Peterson et al. (16) reported that there was no relation between endometriosis and history of menstrual cycle (i.e. length of the cycle, frequency of menstrual cycles per month, and age at menarche).

Use of contraception, as oral contraceptive pills (OCPs) and intrauterine contraceptive device (IUD), is also known to affect menstrual flow. If retrograde menstruation is involved in induction of endometriosis, usage of IUD (a common reason of menorrhagia) would be expected to increase the risk of the disease. Hughes et al. (17) mentioned that the use of IUD not influence the development of endometriosis. In other studies, OCPs exposure was associated with a lower risk of endometriosis (18). Our results indicate no significant difference between usage of contraceptive methods and adhesions with endometriosis in infertile women.

Adnexal area, anterior abdominal wall, bladder and uterus were the most common locations of adhesions in our research. These findings are different from several reports showing that adhesions are more often found in the omentum (19-21). Noticed difference can be due to the point that all cases in our study were women with endometriosis and the most affected areas of endometriosis are the ovaries followed by Douglas’ pouch, uterosacral ligament, vesico-uterine pouch, serosal surface of the uterus, fallopian tubes, round ligament and rectovaginal septum (22). 

In the current study, concerning the associations between endometriosis-related adhesions and quality of life, the results showed that the quality of life is significantly impacted by adhesions. In our study, it was noticed that all the cases with a severe degree of adhesions presented with poor quality of life. This finding was consistent with previous studies (23-25) which discussed that endometriosis and adhesions have significant negative effects on sexual intercourse, educational, social, and familial and professional aspects of the daily life of the patient. The pain, as well as mental and social dysfunction subsequently impairs the quality of life and lowers productivity of working women. Absolute cause or cure is not identified, so this disorder is considered to be chronic and recurrent. The suspected impact of such diseases on sexual relation and fertility has a persistent negative effect on patient’s partnership.

This research had some limitations such as a decrease in the number of participants and samples' readiness in participating. Pain was not discussed in the present study, although it might affect the quality of life as we focused on the adhesions in cases of endometriosis. No long-term follow-up was done in this research as many of patients did not return to the hospitals after doing the procedure of laparoscopy.

## Conclusion

Based on the findings of the present study, it can be concluded that the prevalence of adhesions associated with endometriosis was 37.6%. Also, an association between adhesions related to endometriosis and quality of life among infertile women was found. Further researches might be conducted to study the same problems in larger populations of the women with long-term follow-up.
